# A DFT Screening of M-HKUST-1 MOFs for Nitrogen-Containing Compounds Adsorption

**DOI:** 10.3390/nano8110958

**Published:** 2018-11-20

**Authors:** Shibiao Zong, Yajing Zhang, Na Lu, Pan Ma, Jianguo Wang, Xue-Rong Shi

**Affiliations:** 1 School of Material Engineering, Shanghai University of Engineering Science, 333 Longteng Road, Songjiang District, Shanghai 201620, China; M050117110@sues.edu.cn (S.Z.); 17854311163@163.com (Y.Z.); 05160003@sues.edu.cn (N.L.); mapan@sues.edu.cn (P.M.); 2 State Key Laboratory of Coal Conversion, Institute of Coal Chemistry, Chinese Academy of Sciences, P.O. Box 165, Taiyuan 030001, China; jgwang@sxicc.ac.cn; 3 Institute of Physical Chemistry, University of Innsbruck, Innrain 80-82, A-6020 Innsbruck, Austria

**Keywords:** HKUST-1, metal substitution, density functional theory calculations, molecular adsorption, dissociation, nitrogen-containing compounds

## Abstract

To develop promising adsorbent candidates for adsorptive denitrogenation, we screened the adsorption of NO, NO_2_, and NH_3_ in 19 M-HKUST-1 (M = Be, Fe, Ni, Cr, Co, Cu, V, Zn, Mo, Mn, W, Sn, Ti, Cd, Mg, Sc, Ca, Sr, and Ba) systematically using first-principle calculations. Of these, four variants of M-HKUST-1 (M = Ni, Co, V, and Sc) yield more negative adsorption Gibbs free energy ΔG_ads_ than the original Cu-HKUST-1 for three adsorbates, suggesting stronger adsorbate binding. Ti-HKUST-1, Sc-HKUST-1, and Be-HKUST-1 are predicted to have the largest NO, NO_2_, and NH_3_ adsorption energies within the screened M-HKUST-1 series, respectively. With the one exception of NO_2_ dissociation on V-HKUST-1, dissociative adsorption of NO, NO_2_, and NH_3_ molecules on the other considered M-HKUST-1 is energetically less favorable than molecular adsorption thermodynamically. The barrier calculations show that the dissociation is difficult to occur on Cu-HKUST-1 kinetically due to the very large dissociation barrier. Electronic analysis is provided to explain the bond nature between the adsorbates and M-HKUST-1. Note that the isostructural substitution of Cu to the other metals is a major simplification of the system, representing the ideal situation; however, the present study provides interesting targets for experimental synthesis and testing.

## 1. Introduction

To develop efficient denitrogenation techniques is extremely important due to the detrimental effects of nitrogen-containing compounds (NCCs) on the human health and environment [1,2].

Among various denitrogenation techniques, adsorptive denitrogenation (ADN) is preferred because of its mild operating conditions. The ADN performance strongly depends on the adsorbent. Compared with traditional adsorbents such as activated carbon, metal organic frameworks (MOFs), a new type of adsorbent with metal-oxide units and organic linkers, have recently attracted attention as a promising adsorbent for nitrogen removal due to their structural flexibility and high sorption capacities [3,4].

Among the hundreds of possible MOFs, HKUST-1 is one of the most investigated in recent years. HKUST-1, also known as Cu_3_BTC_2_ or MOF-199, (BTC = 1,3,5 benzene tricarboxylate) has been applied for various purposes: catalysis [5], membranes [6,7], wastewater treatment [8,9], and capturing a wide variety of gases [10]. The gravimetric density of under-coordinated “open-metal” sites in HKUST-1are regarded as the main reaction sites for molecule adsorption [11,12,13]. Xiao et al. [13] found that the NO adsorption capacity of HKUST-1 is as high as 9 mmol g^−1^ at 196 K and 1 bar pressure, which is higher than that of the previously reported porous materials. Infrared spectroscopy experiments show that NO is adsorbed on the coordinatively unsaturated Cu site of HKUST-1. Levasseur et al. [14] prepared GO (graphite oxide) and HKUST-1 composites and conducted NO_2_ adsorption studies under dry and humid conditions. Their results showed that compared with a single adsorbent, only under dry conditions, the composite adsorbent increased the adsorption capacity of NO_2_ significantly. The researchers therefore speculated that in humid conditions, H_2_O formed a competitive adsorption with NO_2_ and thus reduced the amount of NO_2_ adsorption. Hinks et al. [3] obtained high storage capacity for NO using different materials: HKUST-1, MOF-74 (Ni, Co), and MIL-53 (Al, Cr). The adsorption ability followed the sequence: Ni-MOF-74 > Co-MOF-74 > HKUST-1 > Cr-MIL-53 > Al-MIL-53 under the same conditions. Borfecchia et al. [15] studied the interaction of NH_3_ with HKUST-1 by a multitude of characterization techniques. They found NH_3_ chemisorbed on the Cu site under the dry condition and induced the distortion framework, however, retained the crystallinity of the material [15]. According to previous research [8,12,13,14,15], it can be found that HKUST-1 exhibits excellent performance in adsorbing three kinds of NCCs (NO, NO_2_, and NH_3_) and preferentially adsorbed sites are the coordinatively unsaturated metal sites.

Besides the high NO/NO_2_/NH_3_ capacity, recent studies show that the isostructural metal-substituted variants of HKUST-1 can be obtained by an isoform of HKUST-1 with other metals formally in place of copper. For example, recent studies have reported the synthesis of isostructural Cr [16], Ni [17], Zn [18], and Mo-substituted [19] variants of the original Cu-HKUST-1.

Based on the above discussion, HKUST-1 with open metal centers has great potential for metal substitution and in the application for ADN. Despite the extensive experimental studies on the adsorption performance of NCCs on HKUST-1, only a few theoretical researches have focused on NCCs’ adsorption behavior in MOFs. A lot of theoretical work has studied the adsorption in various MOFs of small molecules such as CO_2_ [20,21,22], SO_2_ [23,24], dibenzothiophene [25], CH_4_ [21,26,27], N_2_ [21], N_2_O [28], H_2_ [21], and noble gas [29]. To our best knowledge, a systematic study on the adsorption of three NCCs (NO, NO_2_, and NH_3_) on 19 metal-substituted variants of HKUST-1 is still missing.

In the work, we focus on the adsorption performance of NCCs, including NO, NO_2_, and NH_3,_ on M-HKUST-1 to investigate the effect of the metal center on the adsorption performance of NCCs on HKUST-1. We screened 19 variants of M-HKUST-1 (M = Be, Fe, Ni, Cr, Co, Cu, V, Zn, Mo, Mn, W, Sn, Ti, Cd, Mg, Sc, Ca, Sr, and Ba). The objective of this work is to screen out the high-performance ADN material from various M-HKUST-1.

## 2. Materials and Methods

HKUST-1 (also known as MOF-199) exhibits a simple cubic structure, containing the Cu(II) paddlewheel units which are linked by 1,3,5-benzene tricarboxylate [30] (Figure S1a). To reduce the computational cost, the 156 atom, rhombohedral, primitive cell of HKUST-1 (Figure S1b) was used. The M-HKUST-1 was generated via isostructural substitution of Cu at all the metal ion sites of HKUST-1. Since the native metal center Cu in HKUST-1 exhibits +2 oxidation state, 18-substituted metal elements (M) potentially with a +2 oxidation state were selected: five alkaline earth metals (Be, Mg, Ca, Sr, and Ba), twelve transition metals (Sc, Ti, V, Cr, Mn, Fe, Co, Ni, Zn, Mo, W, and Cd), and one Group IVA metal (Sn). Synthesis of microporous Be(Ba)-based MOFs is not easy in experiments but is nonetheless possible. Kang et al. [31] succeeded to synthesize Be-based MOFs, including Be_2_(OH)_2_(bdc) (BCF-3) and Be_4_(OH)_4_(btec) (BCF-4) (where bdc is 1,3-benzenedicarboxylate and btec is 1,2,4,5-benzenetetracarboxylate) under hydrothermal conditions.

We performed periodic density functional theory (DFT) calculations as implemented in Vienna ab-initio Package (VASP) [32,33] using the projector augmented wave method (PAW) [34] and the Perdew−Burke−Ernzerhof (PBE) [35] generalized gradient approximation of the exchange-correlation functional. Note that a series of previous theoretical studies demonstrated that dispersion-corrected functionals, such as DFT-D2, DFT-D3, and revPBE-vdW, provide the adsorption enthalpies in good agreement with the experimental value upon CO_2_ adsorption on CUS-MOFs [36,37,38]. While CO_2_ yields physical adsorption on CUS-MOFs, the interaction between NCCs molecules and M-HKUST-1 in our studies is mainly chemisorption [15]. Moreover, in this work, we are interested in the trend more than the absolute values, and previous studies [38] show that the PBE functional is good enough to describe the adsorption trend on various MOFs. All calculations are spin-polarized and the cutoff energy of basis set is 540 eV [39]. Due to the large size of the unit cells, only the gamma (1 × 1 × 1) point was used. The force tolerance on each atom between ion steps is relaxed to 0.03 eV Å^−1^. The climbing image nudged elastic band (CI-NEB) [40] method and dimer [41] algorithm are used to search for transition states. Seven geometry images in total are used for each CI-NEB cycle. We calculated the imaginary frequency to verify the transition state that has only one imaginary frequency.

For molecular adsorption, the adsorption energy at 0 K ∆E_ads_ is calculated by:

∆E_ads_ = E_total_(MOFs+N) − E_total_(MOFs) − E_total_(N) (1)

Here, E_total_(MOFs + N), E_total_(MOFs), and E_total_(N) refer to the total energies of the MOFs with the adsorbed nitrogen-containing compounds, the empty MOFs, and the nitrogen-containing compounds in the gas phase, respectively. For dissociative adsorption, here the dissociative adsorption energy at 0 K ∆E_ads-sep_’ is calculated by:

∆E_ads-sep_’ = E_total_(MOFs + N_x1_) + E_total_(MOFs + N_x2_) + … + E_total_(MOFs + N_xn_) − (n − 1)E_total_(MOFs) − E_total_(MOFs + N) (2)

where the dissociated parts are at the infinite separation. E_total_(MOFs + N_xn_) is the total energy of the adsorbed state where N_xn_ denotes the dissociation of the adsorbed molecule into *n* parts.

## 3. Results and Discussion

### 3.1. Bulk Structure

We first performed a geometric optimization of the bulk structure of 19 M-HKUST-1. The calculated Cu–Cu distance in HKUST-1 is 2.46 Å, which is close to the previously calculated value of 2.52 Å and shorted than the corresponding experimental value of 2.58 Å [15]. The data in Table S1 show that the calculated lattice constants of the obtained variant structures are close to the available experimental values, smaller than 1.6%.

According to the structure in the secondary building unit (SBU, Figure S1c), 14 M-HKUST-1 structures (M = Be, Fe, Ni, Cr, Co, V, Zn, Mo, Mn, W, Ti, Cd, Mg, and Sc) of the 18 alternative metal-substituted variants retain the original structure with the distortion degree smaller than 36% (most smaller than 20%), indicating the isostructural metal alternatives is possible. Four M-HKUST-1 variants (M = Sn, Ca, Sr, and Ba) have undergone a distortion with enlarged M–M distance, but retain the original crystal prototype of HKUST-1. The corresponding M–M distance is in the range of 3.54–4.19 Å. Similar results were obtained by Koh et al. [36] from the revPBE-vdW functional. They also found M-HKUST-1 (M = Sn, Ca, and Sr) shows the largest distortion among their screened 18 metal substituted structures but still retains the original prototype structure. Connecting with the adsorption energy of NCCs on M-HKUST-1, the M-HKUST-1 (M = Sn, Ca, Sr, and Ba) variants with the larger distortion exhibit a poor adsorption property among all screened structures. The metal center and its four bonded oxygen atoms in M-HKUST-1 (M = Sn, Ti, Cd, Mg, Sc, Ca, Sr, Ba) exhibit a square-pyramidal-like coordination. The corresponding dihedral angles between plane of M–O1–O2 and the four-fold oxygen plane are larger than 9.5° and the distances from the metal ion to the basal plane of O atoms d(M–4O) are larger than 2.5 Å. While the others exhibit a square-planar-like coordination with a corresponding dihedral angle smaller than 5.7° and d(M–4O) smaller than 1.4 Å. In particular, Sn-HKUST-1 and Ba-HKUST-1, square-pyramidal-like configurations, yield the largest M-M distances of 4.09 and 4.17 Å, respectively, and exhibit the worst adsorption behavior among the 19-screened structures.

### 3.2. Molecular Adsorption

According to the previous experimental [12,13] and theoretical studies [23], the main reaction sites for NO, NO_2_, and NH_3_ adsorption are under-coordinated “open-metal” sites. Chen et al. [23] studied the contribution of each fragment of metal-organic frameworks (MOFs), including HKUST-1 (MOF-199), to the adsorption of sulfur compounds (CH_3_CH_2_SH, CH_3_SCH_3_, and H_2_S) using density functional theory (DFT). They found MOFs with coordinatively unsaturated sites (CUS) have the strongest binding strength with sulfur compounds, and the organic ligands without substituent group has the weakest adsorption strength. Therefore, in the present study, only the under-coordinated “open-metal” sites are considered and the sites of the MOF ligands are ignored. Table 1 presents the adsorption energy and selected geometric parameters for the molecular adsorption of NO and NH_3_ molecules in M-HKUST-1.

#### 3.2.1. NO Adsorption

NO adsorption on the metal site of M-HKUST-1 is via either its O end or N end (Figure S2). The adsorption via the N end of the NO molecule yields ∆E_ads_ of −26.8 kJ mol^−1^, which is close to the previously calculated value, −21.3 kJ mol^−1^ [37], with formed M-N bond distance of 1.97 Å. The adsorption via the O end of NO molecule yields a higher adsorption energy of −11.09 kJ/mol with a larger M-O bond distance of 3.07 Å. The calculations show that NO adsorption on the Cu site of Cu-HKUST-1 by forming the M-N bond is more stable than by forming the M-O bond. Similar results have been observed for the other 18 alternative metal-substituted variants. NO adsorption on the M-HKUST-1 via its N end is more stable than that via its O end. The strongest adsorption via t2 mode yields the adsorption energy of −118.8 kJ mol^−1^ on Sc-HKUST-1, followed by the Ti-HKUST-1 with E_ads_ of −108.5 kJ mol^−1^. This is consistent with our previous observation about NO adsorption on the β-Mo_2_C(0001) surface [42]. NO preferred to adsorb on β-Mo_2_C(0001) via its N end compared to its O end.

The calculated M-N bond distance in t1 mode is between 1.66 Å on Mn-HKUST-1 and 4.11 Å on Sn-HKUST-1 in this study (Table 1). According to the M-N-O angle, the adsorption configurations can be divided into three groups: linear adsorption with the M-N-O angle of ~180°, NO_2_-like adsorption with the M-N-O angle of ~125° which is close to 134° (the O-N-O angle value of NO_2_ in the gas phase), and a bent adsorption with the M-N-O angle of ~155°. NO adsorption on M-HKUST-1 (M = Be, Fe, V, Mn, W, Sn, Ti, Mg, Ca, Sr, and Ba) via t1 mode results in the linear adsorption mode; NO adsorption on Cr-HKUST-1 and Sc-HKUST-1 yields the M-N-O angle of ~155°. Similar to the case on Cu-HKUST-1, NO adsorption via its N end on M-HKUST-1 (M = Ni, Co, Zn, Mo, and Cd) yields the NO_2_-like adsorption mode. The adsorption energy of the MOFs structure ranges from −0.9 kJ mol^−1^ for Sn-HKUST-1 to −236.9 kJ mol^−1^ for Ti-HKUST-1. From the thermodynamic point of view, among the above 19 MOFs structures, twelve alternative M-HKUST-1 (M = Ti, Mn, Sc, Ni, Co, V, Fe, W, Cr, Zn, Cd, and Mg) have the potential to exhibit a stronger NO adsorption capacity than the original Cu-HKUST-1.

#### 3.2.2. NH_3_ Adsorption

For NH_3_ adsorption, we only consider one type of adsorption configuration: top adsorption on the metal center of M-HKUST-1 through the N end of the NH_3_ molecule (Figure S3).

The adsorption of the NH_3_ molecule on the Cu center of HKUST-1 yields the adsorption energy of −79.8 kJ mol^−1^ with the formed M-N bond distance of 2.20 Å.

The MOFs synthesized from the metal centers Be, Zn, Mg, Cd, Co, and Ni have much larger adsorption energy and shorter M-N bond length than Cu-HKUST-1 upon NH_3_ adsorption. Among them, Be-HKUST-1 exhibits the best adsorption performance with the largest adsorption energy ∆E_ads_ of −136.7 kJ mol^−1^ and shortest M-N bond distance of 1.75 Å. Compared with the M-N distance of 4.09 Å and the adsorption energy of −0.8 kJ mol^−1^ on Sn-HKUST-1, the superiority of its adsorption performance for NH_3_ was more prominent. In addition, when Ti-HKUST-1 exhibits excellent adsorption performance for NO, it also shows good adsorption performance for NH_3_, but it is less favorable than Cu-HKUST-1 with adsorption energy of −66.9 kJ mol^−1^, which is about 13 kJ mol^−1^ higher than that of the latter.

#### 3.2.3. NO_2_ Adsorption

For NO_2_ adsorption, we considered four possible adsorption geometries: top adsorption through the N end of NO_2_ molecule (t1), top adsorption through the O end of NO_2_ molecule (t2), bridge adsorption with two oxygen atoms binding to one open metal site (b1), and bridge adsorption with one N and one O atom binding to one open metal site (b2). Table 2 and Table S2 list the adsorption energy and selected geometric parameters.

The most stable adsorption configuration for NO_2_ molecular adsorption on the original Cu-HKUST-1 is the t2 mode by forming one M-O bond. The corresponding M-O bond distance and adsorption energy is 2.24 Å and −40.8 kJ mol^−1^, respectively. The t1 mode by forming one M-N bond yields a much higher adsorption energy of −13.2 kJ mol^−1^. All attempts to get b1 and b2 structure convert to the t2 and t1 modes.

Our calculations show that the strong interaction between the metal center and N(O) end in NO_2_ yields a significant reduction of the N-O bond near the substituted metal center which weakens the N-O bond strength within the NO_2_ molecule (see Figure S4). As a result, the N-O bond in adsorbed NO_2_ structure is observed to undergo elongation. We found two N-O bond lengths of the adsorbed NO_2_ in the t1 and b1 modes are identical, while in the t2 and b2 modes the N-O bond distances near the substituted metal center are shorter than those away from the substrate. The N-O bond length ranges from 1.21 Å on Be-HKUST-1 to 1.25 Å on Ba-HKUST-1 in t1 mode. While in the t2 mode, the N-O bond length near the bonded metal center yields the range from 1.24 Å on Cu-HKUST-1 to 1.42 Å on Sn-HKUST-1 and the N-O bond length away from the substrate gives the range of 1.21–1.24 Å. The average differences between two N-O bond distances are 0.08 Å and 0.09 Å for t2 and b2 modes, respectively.

The calculations show that the NO_2_ adsorption on alkaline earth metal substituted variants follows the sequence: Be-HKUST-1 < Mg-HKUST-1 < Ca-HKUST-1 < Sr-HKUST-1 < Ba-HKUST-1, while the NH_3_ adsorption exhibits different adsorption behavior towards the substitution of Cu center by the alkaline earth metal. The adsorption ability follows the sequence in the reverse order: Be-HKUST-1 > Mg-HKUST-1 > Ca-HKUST-1 > Sr-HKUST-1 > Ba-HKUST-1. Among these, the energetically most favorable center is Be-HKUST-1, with the lowest adsorption energy of −136.7 kJ mol^−1^.

**Figure 1:**
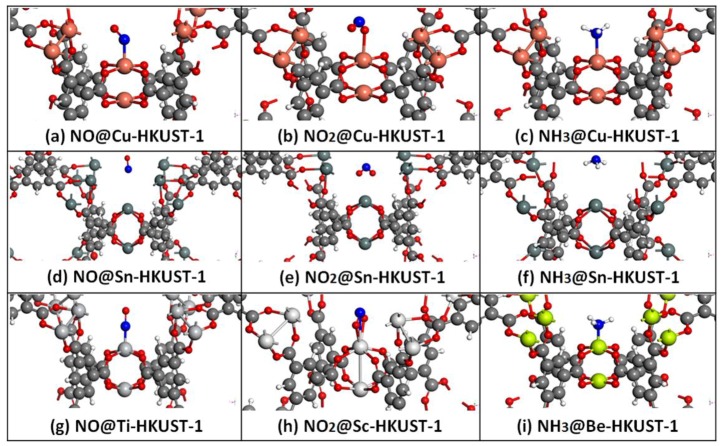
Representative adsorption structures near the secondary building units (SBUs) on selected metal organic frameworks (MOFs): (**a**) NO on Cu-HKUST-1, (**b**) NO_2_ on Cu-HKUST-1, (**c**) NH_3_ on Cu-HKUST-1, (**d**) NO on Sn-HKUST-1, (**e**) NO_2_ on Sn-HKUST-1, (**f**) NH_3_ on Sn-HKUST-1, (**g**) NO on Ti-HKUST-1, (**h**) NO_2_ on Sc-HKUST-1, and (**i**) NH_3_ on Be-HKUST-1. The largest spheres are metal atoms. White, red, blue, and grey spheres are H, O, N, and C atoms with increasing size, respectively.

Based on the above description, Ti-HKUST-1 exhibits the best adsorption performance for NO adsorption, and it also shows an excellent adsorption behavior towards NO_2_ adsorption, which is better than Cu-HKUST-1 and only worse than Sc-HKUST-1. Sc-HKUST-1 is found to yield the best adsorption performance upon NO_2_ adsorption; see Figure 1. While for NH_3_ adsorption, Ti-HKUST-1 shows a slightly weaker adsorption property than Cu-HKUST-1. Instead, Be-HKUST-1 has the best adsorption effect for NH_3_. It should be noted that M-HKUST-1 (M = Ni, Co, V, and Sc) exhibits a better adsorption performance than Cu-HKUST-1 for three NCCs. The adsorption performance of Sn-HKUST-1 on the above gases is poor. As shown in Table S1, Table 1, and Table 2, when the structure of the M-HKUST-1 is deformed, it has relatively smaller adsorption energy, resulting in a poor adsorption performance.

### 3.3. Dissociative Adsorption

Since the original metal center of HKUST-1 is Cu, we first investigate the dissociation adsorption of NCCs on Cu-HKUST-1.

Considering the separation adsorption of nitrogen and oxygen at infinite position on HKUST-1, the dissociation energy ∆E_ads-sep_’ of NO is 586.9 kJ mol^−1^, suggesting the dissociative NO adsorption is strongly endothermic. The dissociative adsorption energy shifts significantly to more positive E_ads_ values compared with the results for molecular adsorption. Therefore, the dissociative adsorption of NO is much less favorable than molecular adsorption thermodynamically suggesting strongly that NO will not dissociate on Cu-HKUST-1. All attempts to obtain the co-adsorption of N and O on the same Cu center resulted in the molecular adsorption of NO. Thus, there was no barrier search for NO dissociation on HKUST-1.

In the process of dissociative adsorption of NO_2_ to yield the products at the separated adsorption, the dissociation energies for the first and second steps are 318.0 and 586.9 kJ mol^−1^, respectively. Both steps are strongly endothermic indicating that the dissociation is unfavorable thermodynamically. Again, all attempts to obtain the co-adsorption of NO and O on the same Cu center resulted in the molecular adsorption of the NO_2_ molecule. Therefore, it can be concluded that NO and NO_2_ have rare possibility of dissociation to yield co-adsorption products on HKUST-1.

**Figure 2:**
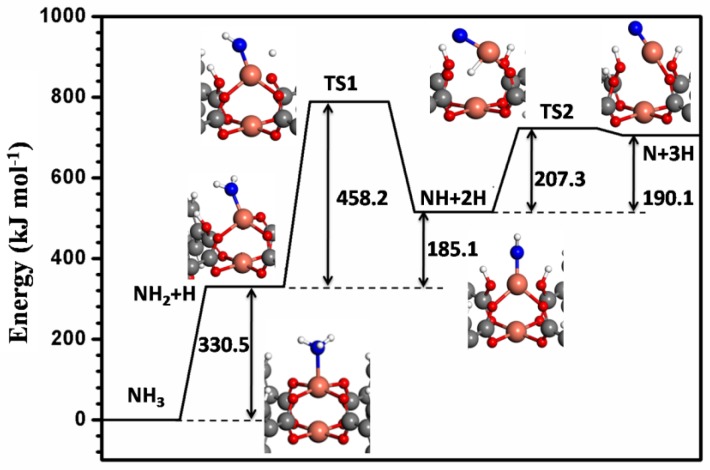
Reaction paths for NH_3_ dissociative adsorption on Cu-HKUST-1. TS1 is the transition state for NH_2_ dissociation into NH + H. TS2 is the transition state for NH dissociation to N + H. Adsorbate geometries present in the local MOFs region. The color scheme is the same as for Figure 1.

The energy profile of reaction paths for NH_3_ dissociation adsorption on Cu-HKUST-1 is provided in Figure 2. The reaction energy ∆E_ads-sep_’ for the dehydrogenation of NH_3_ to yield NH_2_ on the metal center and H on the oxygen atom in the absence of any lateral interactions was found to be strongly endothermic by 279.9 kJ mol^−1^. In the most stable co-adsorption configuration of hydrogen and NH_2_, NH_2_ adsorbs on the open Cu center while H prefers the neighboring oxygen center, which results in the cleavage of the O-Cu bond. Consequently, the reaction energy changed to 330.5 kJ mol^−1^, indicating the reaction is still strongly endothermic. A further dehydrogenation of NH_2_ to produce NH and H yields a large reaction barrier of 458.2 kJ mol^−1^. The corresponding reaction energy is 192.8 kJ mol^−1^ with NH and H at infinite separation. When NH_2_ adsorbs on the open Cu center and H co-adsorbed on the neighboring oxygen center, the reaction energy changed to 185.1 kJ mol^−1^. The final dehydrogenation of NH to N and H is connected with a reaction barrier of 207.3 kJ mol^−1^ and dissociation energy of 190.1 kJ mol^−1^. As described above, the reaction energy and barrier for all dissociative adsorption processes are high; therefore, the possibility of dissociative adsorption is essentially eliminated thermodynamically and kinetically.

Based on the discussion in Section 3.1, we then chose 11 out of 19 candidates to study the dissociative adsorption, as provided in Table 3. Among them, Ti-HKUST-1, Sc-HKUST-1, and Be-HKUST-1 exhibit the most stable NO, NO_2_, and NH_3_ molecular adsorption, respectively. M-HKUST-1 (M = Sn, Cd, Ba) exhibits the least stable NCCs molecular adsorption and M-HKUST-1(M = Ni, Co, and V) yields a better adsorption effect than Cu-HKUST-1 for three NCCs. Fe-HKUST-1 is also picked due to its excellent adsorption performance for NO and NO_2_ adsorption. To study the dissociation, we first investigate the adsorption of the dissociated species, e.g., NH_x_ (x = 0–2), atomic H and atomic O. The calculations show that O binds to the metal sites of M-HKUST-1 more strongly than NH_x_ (x = 0–2) species with larger adsorption energies suggesting that atomic oxygen will compete with the dissociated NH_x_ species for the open metal sites strongly and will block NH_x_ adsorption. As shown in Table 3, the dissociative adsorption yields the energy ranges (in kJ mol^−1^) of −147.1 to 649.8, −174.9 to 355.6, and −2.9 to 351.0 for NO, NO_2_, and NH_3_ adsorption, respectively. It shifts to more positive ∆E_ads_ values (in kJ mol^−1^) compared with the corresponding values for molecular adsorption with −236.9 to −0.9, −303.6 to −11.4, and −136.7 to −0.8, respectively. This means that dissociative adsorption is less likely to happen compared with the molecular adsorption thermodynamically. However, there is one exception: NO_2_ adsorption on V-HKUST-1 prefers to dissociate into NO and O, and the yielded NO will retain the molecular adsorption with no further dissociation into N and O thermodynamically.

Overall, except for NO_2_ adsorption on V-HKUST-1, the dissociative NO, NO_2_, and NH_3_ adsorption is less able to happen compared with the molecular adsorption thermodynamically and kinetically.

### 3.4. Thermodynamics

The NCC adsorption process on MOFs is described by the following equilibrium reaction:

MOFs + NCCs(gas) ↔ NCCs-MOFs(3)

Similar to our previous work [43], the corresponding adsorption Gibbs free energy, ∆G_ads_(T, P), is calculated by

∆G_ads_(T, P) = ∆E_ads_ − G^θ^(T) − RTln(P_NCCs_/P^θ^) (4)

where ∆E_ads_ is the adsorption energy at 0 K, and G^θ^(T) represents the thermo items including the contributions from translation, rotation, and vibration of the gas phase NCCs.

**Figure 3:**
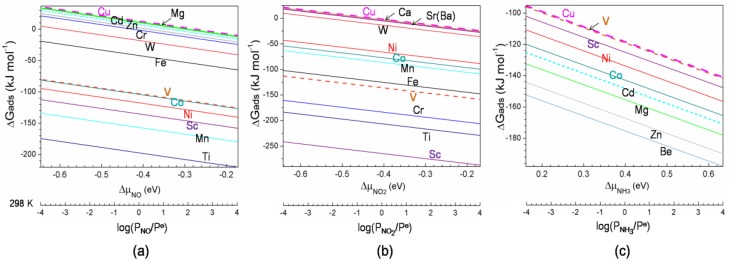
Adsorption Gibbs free energy, ∆G_ads_(T, P) of (**a**) NO, (**b**) NO_2_, and (**c**) NH_3_ adsorption on various M-HKUST-1 as a function of the nitrogen-containing compound (NCC) chemical potential, where μ_NCCs_ is defined as G^θ^(T) + RTln(P_NCCs_/P^θ^) including zero-point vibrational energy. The corresponding pressure is provided for the selected temperature. To make the picture clear, only those with larger ∆G_ads_(T, P) than Cu-HKUSU-1 are included in the figure.

**Figure 4:**
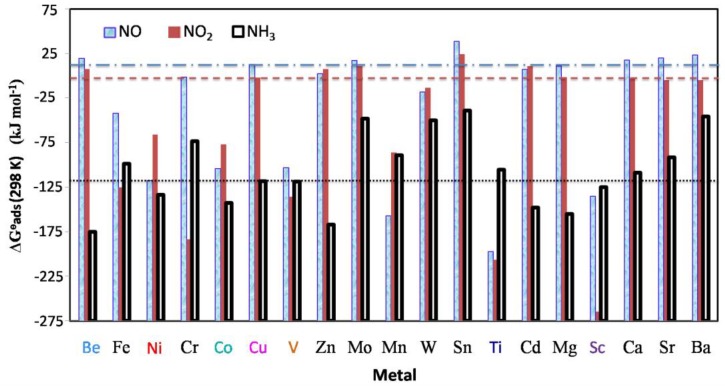
Standard adsorption Gibbs free energy ∆G^θ^_ads_ (298 K) of NO, NO_2_, and NH_3_ adsorption on metal substituted variants of HKUST-1 with the partial pressure of NCCs at 1 atm. The blue dash dot/red dash/dark dot line corresponds to the ∆G^θ^_ads_ (298 K) value of NO/NO_2_/NH_3_ adsorption on Cu-HKUST-1. The shorter the bar, the stronger the binding.

The corresponding results are shown in Figure 3 and Figure 4. In Figure 3, we provide adsorption Gibbs free energy of NO, NO_2_, and NH_3_ adsorption on various M-HKUST-1 as a function of the NCC chemical potential. The NCC chemical potential is correlated with pressure for room temperature (RT) as is characteristic of real temperatures employed in experiments [13,44,45].

It can be seen that twelve metal-substituted variants are predicted to be more suitable for NO adsorption than the original Cu-HKUST-1 in a pressure region of 10^−4^–10^4^ atm at 298 K. The metal center yields the stability of NO adsorption in a decreased sequence: Ti > Mn > Sc > Ni > Co ≈ V > Fe > W > Cr > Zn > Cd > Mg ≈ Cu. The NO adsorption on Cu-HKUST-1 becomes favorable thermodynamically when NO chemical potential is larger than −0.24 eV, corresponding to NO pressure higher than 165 atm at 298 K.

Twelve (eight) variants of M-HKUST-1 have the potential to exhibit better performance for NO_2_ (NH_3_) capture than Cu-HKUST-1. The corresponding stability of NO_2_ adsorption on M-HKUST-1 decreased in sequence: Sc > Ti > Cr > V > Fe > Mn > Co > Ni > W > Sr ≈ Ba > Ca > Cu. For NH_3_ adsorption, the sequence changed to Be > Zn > Mg > Cd > Co > Ni > Sc > V ≈ Cu. The thermodynamical analysis also shows that the NO_2_ adsorption on Cu-HKUST-1 becomes exothermic with the NO_2_ partial pressure higher than ~0.5 atm at 298 K connected with NO_2_ chemical potential larger than −0.38 eV. NH_3_ adsorption on all considered M-HKUST-1 is exothermic in our considered NH_3_ chemical potential range indicating it is always favorable thermodynamically in our considered range.

Adsorption Gibbs free energies on 19 M-HKUST-1 (Figure 4) shows that five alkaline earth metals together with Zn, Mo, and Sn show the same trend with Cu-HKUST-1 towards three NCCs adsorption and the stability order is: NH_3_ > NO_2_ > NO. A similar result was observed by Supronowicz et al. [11] who found NH_3_ bonds to HKUST-1 stronger than NO_x_ by DFT calculations.

### 3.5. Analysis of Electronic Properties

Adsorption of NCCs on M-HKUST-1 is associated with charge transfer between the MOFs and the adsorbate. To understand the nature of chemical bonding between NCCs and the M-HKUST-1 substrate, we perform the Bader charges [46] analysis, partitioning a charge density grid into Bader volumes scaled linearly with the number of grid points on individual atoms. Table 4 provides the net NCC charge upon adsorption.

As shown in Table 4, NO_x_ (x = 1, 2) adsorption results, for almost all M-HKUST-1, in a negatively charged adsorbate species indicating adsorbates accepts electrons from the MOFs while the opposite trend is found for NH_3_ adsorption where NH_3_ is positively charged suggesting the electrons transfers from NH_3_ to the MOFs. The negative charging indicates the number of back-donated electrons to the antibonding orbitals of the NO_x_ (x = 1, 2) adsorbates is larger than that of denoted electrons from adsorbates to the MOFs. This results in the weaker interatomic bonds and increased interatomic distances d_N-O_ (Table 1). The similar result is observed for NO adsorption on β-Mo_2_C in our previous work [42]. We found NO are negatively charged on the β-Mo_2_C(0001) surface [42], while the NH_3_ molecule, as a typical Lewis base with a lone pair of electrons, prefers to denote elections to the substrate during the adsorption. We also found that the charge values of adsorbed NO_2_ are larger than those of adsorbed NO and NH_3_ in absolute size indicating a larger charge transfer between NO_2_ and MOFs than the other two systems.

**Figure 5:**
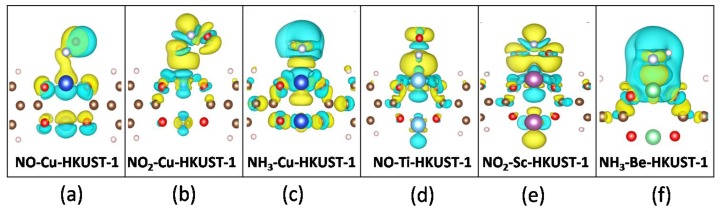
Selected electron density difference map for NCC molecules adsorbed on M-HKUST-1: (**a**) NO on Cu-HKUST-1, (**b**) NO_2_ on Cu-HKUST-1, (**c**) NH_3_ on Cu-HKUST-1, (**d**) NO on Ti-HKUST-1, (**e**) NO_2_ on Sc-HKUST-1, and (**f**) NH_3_ on Be-HKUST-1. For each case, only the most stable adsorption configuration is considered. Accumulation region in yellow and depletion regions in blue.

We then examined the distribution of charge for NCC molecular adsorption on M-HKUST-1 (M = Cu, Ti, Sc, and Be) using electron density difference maps. The electron density difference (∆ρ) was calculated according to our previous work [47]. The charge density maps are also provided in Figure S5 for comparison; note that non-covalent interaction plots [48] may provide extra information about the inter and intramolecular non-covalent interactions (i.e., hydrogen bonds, steric clashes and van der Waals) in systems, while the electronic analysis based on the Bader charge analysis and electron density difference maps in this work can provide enough data regarding the nature of the chemical bonding. As shown in Figure 5, some metal orbitals are found to be depleted upon adsorption on MOFs associated with the charge redistribution of the M–O bond in the SBUs and new formed M–N/O bond. When the electron density of the M–O bond in the SBUs decreased, the electron density along the M−N/O bond increased. Compared with the original Cu-HKUST-1, the change of the electron density of the M−N bond is larger for M-HKUST-1 (M = Ti, Sc, and Be).

### 3.6. Correlations

#### 3.6.1. Adsorption Energy and Bader Charge

**Figure 6:**
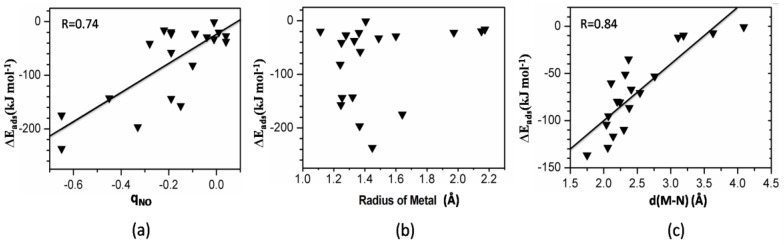
The calculated relationship between the (**a**) NO adsorption energy on M-HKUST-1 and total Bader charge on adsorbed NO, (**b**) NO adsorption energy and metal atomic radius, and (**c**) NH_3_ adsorption energy and M-N bond distance.

The correlation between the adsorption energy and the Bader charge of the adsorbed molecules, the metal atomic radius, and the forming M-N band distance is provided in Figure 6.

In general, the adsorption energy is linearly related to the total Bader charge of the adsorbed molecule since the electrostatic interaction is an important part of the interaction between the MOFs and the NCCs molecules. We take the NO_2_ adsorption on M-HKUST-1 as an example. As shown in Figure 6a, the most charged compounds in the adsorbed molecules exhibited the largest adsorption energy. NO_2_ adsorption on Sc-HKUST-1 via the b1 mode with the largest adsorption energy yields the largest Bader charge of −0.77 among all the adsorbed NO_2_ species. Similarly, the adsorbed NO on Ti-HKUST-1 presents the largest Bader charge of −0.65 resulting in the strongest adsorption.

#### 3.6.2. Adsorption Energy and Metal Radius

Figure 6b depicts the relationship between the adsorption energy and the atomic radius of the substituted metal atom. Generally, the smaller the metal atom, the stronger the polarization effect, and the stronger polarization effect will induce a stronger binding between the adsorbates and MOFs. Our results show that no clear dependence between the adsorption energy and the metal atomic radii was observed in these compounds. This behavior can be understood. Besides the polarization effect, the substitution by the metal atom with different atomic radius also has the size effect, which will affect the structure of the SBU. A case in point is NCC molecular adsorption on Be-HKUST-1. As an element with the smallest atomic radius in our studies, it is expected that Be has the strongest polarization effect on NCC molecules, resulting in the largest ΔE_ads_. It is only true for NH_3_ adsorption where Be-HKUST-1 yields the largest ΔE_ads_ towards NH_3_ adsorption; for NO and NO_2_ adsorption, this is not the case. The actual adsorption energy for NO_2_ (NO) adsorption is only −2.9 (−20.0) kJ mol^−1^, which is 38 (6.8) kJ mol^−1^ higher than that of Cu-HKUST-1. This can be attributed to the size/structural effect within the SBU. With the smaller radius, when Be employs the same coordination pattern as the Cu-HKUST-1 prototype, it will induce a strong contraction of the MOFs structure, especially within the SBU with the much shorter d(M-O) of 1.74 Å. As a result, the most stable adsorption configuration is unpredictable. For NO_2_ adsorption, the energetically favorable structure shifts from the t1 with the central N atom binding to the metal center to t2 with the oxygen end binding to the metal center. In general, the relationship between the NCCs adsorption energy and the metal radius is complicated by changes in the structure of the MOFs due to the size/structural effects. The same phenomenon has been observed for CO_2_ adsorption HKUST-1 by Hyun et al. [36].

#### 3.6.3. Adsorption Energy and M-N(O) Bond Distance

Interestingly, we found a linear relationship between the adsorption energy and the forming M-N band distance for NH_3_@M-HKUST-1 (Figure 6c), while, for the other systems, there is a clear suggestion that the linear relationship is broken by the low-symmetry of adsorbed structure upon the adsorption of NO and NO_2_ on MOFs. For example, the formed M-N-O angle towards NO adsorption on M-HKUST-1 divided into three groups generally: ~180°, ~125°, and ~155° suggesting strongly the broken symmetry of M-HKUST-1 and inducing a low symmetry of the adsorbed states. Therefore, this linear model has limitations and should be carefully used to explain adsorption trends of adsorbates on MOFs.

## 4. Conclusions

Metal-organic frameworks show great potential for toxic gas capture applications due to their high adsorption capacities and structural flexibility. Among them, metal-substituted M-HKUST-1 exhibits very high NCC uptake at low pressures due to its gravimetric density of under-coordinated “open-metal” sites. To identify promising candidates with better ADN performance than those currently known ones, we use first principle calculations to screen 19 metal-substituted compounds M-HKUST-1 (M = Be, Fe, Ni, Cr, Co, Cu, V, Zn, Mo, Mn, W, Sn, Ti, Cd, Mg, Sc, Ca, Sr, and Ba) towards their NCC adsorption behavior.

Our calculations show that among 19 screened M-HKUST-1, four M-HKUST-1 variants (M = Ni, Co, V, and Sc) with excellent adsorption properties for three nitrogen-containing compounds (NO, NO_2_, and NH_3_) were screened out. Their adsorption capacities for three nitrogen-containing compounds are expected to exceed their original structure (Cu-HKUST-1). Considering a specific adsorption target separately, Ti-HKUST-1 shows the best adsorption performance for NO with the largest adsorption energy of −236.9 kJ mol^−1^. For NO_2_ adsorption, Ti-HKUST-1 also shows an excellent performance, but Sc-HKUST-1 is even better. Sc-HKUST-1 is found to yield the strongest interaction between NO_2_ molecule and MOFs with ∆E_ads_ of −303.6 kJ mol^−1^. For NH_3_ adsorption, Be-HKUST-1 yields the largest NH_3_ adsorption energy of −136.7 kJ mol^−1^, which is 56.9 kJ mol^−1^ L larger than Cu-HKUST-1, suggesting the best NH_3_ adsorption behavior.

The dissociative adsorption for all NCCs is much less favorable compared with molecular adsorption thermodynamically. There is only one exception. NO_2_ adsorption on V-HKUST-1 prefers to dissociate into NO and O thermodynamically. A further calculation for NH_3_ dissociation on the original Cu-HKUST-1 connecting the energetically most stable co-adsorption geometries yields a reaction barrier as high as 458.2 kJ mol^−1^, which is consistent with the experimental observation that upon NH_3_ adsorption on Cu-HKUST-1, no adsorbed NH_3_ undergoes decomposition.

The bulk calculations show that extremely small or large ions will distort the structure of HKUST-1, causing structural effects. As a result, the adsorption energy of NCCs on M-HKUST-1 is not related to the ionic radii of the substituted metals. The lateral electronic analysis found a linear relationship between the total Bader charge of the adsorbed NCC molecules and adsorption energy. These results indicated the adsorption of NCCs on M-HKUST-1 mainly relies on the electronic structure instead to depend on the structural/size features of the MOFs. However, a simple model of net charge transfer cannot be employed to all of the NCCs species in this work. NO and NO_2_ on M-HKUST-1 are negatively charged, while the absorbed NH_3_ is positive. Despite the fact that the isostructural substitution of Cu to the other metals is a major simplification of the system that represents the ideal situation, and the synthesized structure in experiments may be much more complex, the present studies may shed light on searching for better adsorbants of ADN for the MOF community.

## Figures and Tables

**Table 1:** Calculated distances between the metal center and the nitrogen d(M-N) (Å), N-O bond lengths within NO d(N-O) (Å), bond angles (Ang.) formed by the metal center of M-HKUST-1 and O and N in NO (°) and adsorption energy ∆E_ads_ (kJ mol^−1^) for molecular adsorption of NO and NH_3_ on M-HKUST-1.

	NO-t1				NO-t2				NH_3_	
	d(M-N)	d(N-O)	Ang.	∆E_ads_	d(M-O)	d(N-O)	Ang.	∆E_ads_	d(M-N)	∆E_ads_
gas	--	1.17	--	--	--	--	--	--	--	--
Be	1.96	1.17	174	−20.0	2.23	1.17	168	−0.4	1.75	−136.7
Fe	1.71	1.18	178	−81.6	1.91	1.19	178	−15.0	2.11	−60.3
Ni	1.81	1.18	122	−157.0	3.30	1.17	140	−3.4	2.07	−95.2
Cr	1.83	1.19	151	−41.0	3.24	1.17	175	−0.9	2.37	−35.0
Co	1.80	1.18	128	−143.6	1.98	1.18	130	−74.1	2.04	−104.3
Cu	1.97	1.17	126	−26.8(−21.31) ^1^	3.07	1.16	157	−11.1	2.20	−79.8
V	1.75	1.20	176	−142.5	2.00	1.20	152	−62.0	2.24	−80.5
Zn	2.21	1.17	129	−37.2	2.50	1.17	179	−12.3	2.06	−128.7
Mo	2.39	1.19	127	−22.4	3.11	1.18	177	−0.8	3.19	−9.8
Mn	1.66	1.18	179	−196.5	1.81	1.19	178	−67.3	2.32	−51.1
W	1.90	1.20	174	−57.6	3.01	1.19	177	−2.4	3.11	−11.9
Sn	4.11	1.17	173	−0.9	4.40	1.17	177	−0.5	4.09	−0.8
Ti	1.82	1.22	179	−236.9	1.92	1.25	180	−108.5	2.41	−66.9
Cd	2.45	1.17	130	−32.6	2.71	1.17	180	−8.8	2.30	−109.6
Mg	2.25	1.17	179	−28.7	2.24	1.18	180	−16.7	2.14	−116.8
Sc	2.04	1.22	159	−174.8	2.04	1.25	149	−118.8	2.38	−86.5
Ca	2.60	1.18	179	−21.9	2.56	1.18	179	−11.8	2.54	−70.6
Sr	2.78	1.19	177	−19.4	2.73	1.19	180	−12.4	2.76	−53.1
Ba	3.04	1.19	177	−16.0	3.04	1.19	180	−0.3	3.63	−7.3

^1^ Ref. [37], DFT value, Turbomole code, hybrid B3LYP functional.

**Table 2:** Calculated adsorption energy ∆E_ads_ (kJ mol^−1^) and metal-nitrogen/oxygen bond length d(M-N/O) (Å) between the center of the metal and nitrogen or oxygen in NO_2_ for molecular adsorption of NO_2_ on M-HKUST-1. The ∆E_ads_ values for the most stable adsorption mode are labeled in bold.

	(t1)		(t2)		(b1)		(b2)	
	d(M-N)	ΔE_ads_	d(M-O)	ΔE_ads_	d(M-O/O’)	ΔE_ads_	d(M-N/O)	ΔE_ads_
Be	2.68	−2.9	1.74	**−31.4**	b1 → t2	--	b2 → t2	--
Fe	2.03	**−164.0**	2.00	−85.1	b1 → t2	--	b2 → t1	--
Ni	1.94	**−105.0**	1.99	−66.6	b1 → t2	--	b2 → t1	--
Cr	t1 → b2	--	2.03	−64.5	b1 → t2	--	1.99/2.31	**−222.5**
Co	1.95	**−115.9**	1.97	−82.1	b1 → t2	--	b2 → t1	--
Cu	2.22	−13.2	2.24	**−40.8**	b1 → t2	--	b2 → t1	--
V	2.15	−82.2	1.89	−116.5	b1 → t2	--	2.07/2.22	**−125.4**
Zn	2.19	−11.3	2.08	**−31.2**	b1 → t2	--	b2 → t2	--
Mo	2.48	−24.1	2.42	**−28.0**	b1 → t2	--	b2 → t1	--
Mn	2.05	−94.5	1.93	**−125.2**	b1 → t2	--	b2 → t1	--
W	2.40	−46.3	2.32	**−52.2**	b1 → t2	--	b2 → t1	--
Sn	3.39	−4.6	2.04	−6.6	2.88/2.94	**−14.5**	b2 → t1	--
Ti	t1 → b2	--	t2 → b1	--	2.20/2.19	−224.0	2.05/1.99	**−245.5**
Cd	2.43	−11.5	2.41	**−27.9**	b1 → t2	--	b2 → t1	--
Mg	2.28	−5.7	2.08	**−40.6**	b1 → t2	--	b2 → t2	--
Sc	t1 → b2	--	2.02	−289.9	2.27/2.27	**−303.6**	2.25/2.12	−295.9
Ca	t1 → t2	--	2.43	**−42.1**	2.63/2.62	−32.9	b2 → t2	--
Sr	t1 → t2	--	2.60	**−43.8**	2.75/2.86	−40.0	b2 → t2	--
Ba	3.02	−33.8	2.85	**−43.9**	2.98/3.06	−43.6	b2 → t2	--

**Table 3:** The dissociative adsorption energy ∆E_ads-sep_’ (kJ mol^−1^) of nitrogen-containing compounds on selected M-HKUST-1.

	NO → N + O	NO_2_ → NO + O	NO_2_ → N + 2O	MH_3_ → NH_2_ + H	NH_3_ → NH + 2H	NH_3_ → N + 3H
gas	734.7	446.1	1180.8	471.2	887.6	1260.2
Be	619.3	329.3	948.6	284.1 ^2^	691.8 ^2^	1221.5 ^2^
Fe	299.8	133.3	433.1	189.1 ^1^	516.2 ^2^	550.0 ^1^
Ni	484.3	123.0	607.3	267.3 ^2^	543.2 ^2^	753.6 ^2^
Co	397.4	99.9	497.3	264.4 ^1^	542.3 ^1^	739.1 ^1^
Cu	586.9	318.0	904.9	279.9 ^2^	472.7 ^2^	662.9 ^2^
V	−139.2	−174.9	−314.1	200.7 ^2^	353.1 ^2^	506.6 ^2^
Sn	336.8	132.5	469.3	268.2 ^2^	581.8 ^2^	946.5 ^2^
Ti	−147.1	−162.4	−309.5	−2.9 ^1^	100.3 ^1^	316.5 ^1^
Cd	649.8	339.7	989.5	351.0 ^2^	667.4 ^2^	927.1 ^2^
Sc	51.8	−36.7	15.1	153.8 ^2^	570.6 ^2^	1031.8 ^2^
Ba	622.9	355.6	978.5	295.2 ^2^	594.3 ^2^	843.3 ^2^

^1^ H at metal center; ^2^ H at the O near the metal center.

**Table 4:** Bader charge q_x_(x = NO, NO_2_-t1, NO_2_-t2, NO_2_-b1, NO_2_-b2, and NH_3_) for NCCs on M-HKUST-1 (The charges are shown for the entire NCCs molecule).

Metal	q_NO_	q_NO2-t1_	q_NO2-t2_	q_NO2-b1_	q_NO2-b2_	q_NH3_
Be	0.01	0.00	−0.20	--	--	0.11
Fe	−0.10	−0.45	−0.55	--	--	0.19
Ni	−0.15	−0.26	−0.38	--	--	0.17
Cr	−0.28	--	−0.27	--	−0.40	0.08
Co	−0.19	−0.37	−0.45	--	--	0.19
Cu	0.04	−0.17	−0.09	--	--	0.11
V	−0.45	−0.49	−0.58	--	−0.53	0.14
Zn	0.04	−0.05	−0.15	--	--	0.18
Mo	−0.19	−0.42	−0.44	--	--	0.03
Mn	−0.33	−0.51	−0.57	--	--	0.13
W	−0.19	−0.56	−0.60	--	--	0.04
Sn	−0.01	−0.08	−0.01	−0.29	--	0.00
Ti	−0.65	--	--	−0.72	−0.72	0.07
Cd	−0.01	−0.10	−0.07	--	--	0.16
Mg	−0.04	−0.32	−0.16	--	--	0.06
Sc	−0.65	--	−0.75	−0.77	−0.73	0.08
Ca	−0.09	--	−0.31	−0.44	--	0.06
Sr	−0.19	--	−0.48	−0.57	--	0.04
Ba	−0.22	−0.52	−0.52	−0.57	--	0.02

## Supplementary Materials

The following are available online at http://www.mdpi.com/2079-4991/8/11/958/s1, Figure S1: (a) HKUST-1 bulk structure containing unsaturated metal sites, (b) bulk structures used in this work, (c) the structure of secondary building unit (SBU), (d) three pores in inactivated HKUST-1, (e) the side view with the similar angle of our used structure b, (f) the zoomed pore which will be occupied by the adsorbed NCCs molecules. Figures S2–S4: Adsorption configuration for NO, NH_3_ and NO_2_ molecular adsorption on M-HKUST-1. Figure S5: The 3D (top) and 2D (bottom) charge density map measured in e/Å3 for (a) NO, (b) NO_2_, and (c) NH_3_ adsorption on Cu-HKUST-1. Table S1: Calculated bulk lattice parameters, metal-oxygen bond length d(M-O) (Å), metal-metal distance d(M–M) (Å), a distance between metal and the basal plane of four O atoms d(M-4O) (Å) of M-HKUST-1, dihedral angle θ (°) formed by one metal center and its three bonded oxygen atoms in SBU, deformation degree (%) and distance between metal atoms M2 and M3 d(M2–M3) (Å) shown in Figure S1. Table S2: The bond distance and bond angle of NO_2_ in the adsorption state.
