# Supplementary material for: A DFT Screening of M-HKUST-1 MOFs for Nitrogen-Containing Compounds Adsorption
Source: Nanomaterials (Basel). 2018 Nov 20;8(11):958. doi: 10.3390/nano8110958 (PMC6266483; doi:10.3390/nano8110958)
Supplement: Supplementary file 1 [file nanomaterials-08-00958-s001.pdf]

Supplementary Materials for

# A DFT Screening of M-HKUST-1 MOFs for Nitrogen-Containing Compounds Adsorption

**Shibiao Zong [1], Yajing Zhang [1], Na Lu [1], Pan Ma [1], Jianguo Wang [2] and Xue-Rong Shi [1,3,*]**

[1] School of Material Engineering, Shanghai University of Engineering Science, 333 Longteng Road, Songjiang District, Shanghai, P. R. China; shixuer05@mails.ucas.ac.cn(X.-R.S.) , M050117110@sues.edu.cn(S.Z.), M050118113@sues.edu.cn(Y.Z.),05160003@sues.edu.cn(N.L.), mapan@sues.edu.cn(P.M.)

[2] State Key Laboratory of Coal Conversion, Institute of Coal Chemistry, Chinese Academy of Sciences, P.O. Box 165, Taiyuan, Shanxi 030001, P. R. China; jgwang@sxicc.ac.cn

[3] Institute of Physical Chemistry, University of Innsbruck, Innrain 80-82, Innsbruck A-6020, Austria

* Correspondence: shixuer05@mails.ucas.ac.cn; Tel.: +86-21-6779-1380

## 1. Model

As shown in Figure S1, HKUST-1 presents three kinds of pores. For NO and $NH_3$ chemi-adsorption on the metal sites, the M1, M2 site in HKUST-1 and N in NCCs form a line which will cross the yellow pore. For $NO_2$ adsorption, the plane formed by M1, M2 and N will cut the yellow pore. M1, M2, M3, and M4 metal atoms are in a line and cross the center of the yellow pore. Therefore, we summarize the distance between M2 and M3 in Table S1 to represent size of the yellow pore.

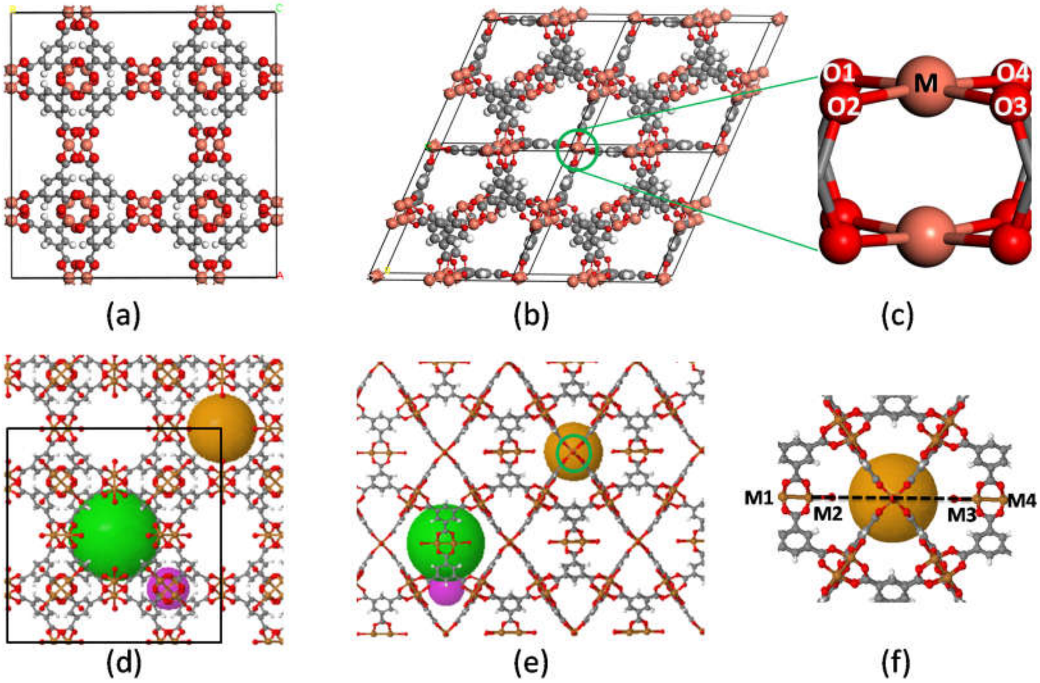


**Figure S1.** (a) HKUST-1 bulk structure containing unsaturated metal sites, (b) bulk structures used in this work, (c) the structure of secondary building unit (SBU), (d) three pores in unactivated HKUST-1, (e) the side view with the similar angle of our used structure b, (f) the zoomed pore which will be occupied by the adsorbed NCCs molecules. M1, M2, M3, and M4 metal atoms are in a line and cross the center of the pore. Small white, red, grey, and largest spheres are H, O, C, and Cu, respectively. The pore figure d-e is from the website of University of Liverpool where Cu atoms in HKUST-1 are saturated by oxygen atoms: http://www.chemtube3d.com/solidstate/MOF-HKUST-1.html.

## 2. Calculated Bulk Structure of M-HKUST-1

**Table S1.** Calculated bulk lattice parameters, metal-oxygen bond length d(M-O) (Å), metal-metal bond length d(M-M) (Å), a distance between metal and the basal plane of four O atoms d(M-4O) (Å) of M-HKUST-1, dihedral angle ɵ (°) formed by one metal center and its three bonded oxygen atoms in SBU, deformation degree (%) and distance between metal atoms M2 and M3 d(M2-M3) (Å) shown in Figure S1. Available experimental values are provided in bracket.

| | a=b=c | d(M-O) | d(M-M) | d(M-4O) | ɵ | def. deg. [a] | d(M2-M3) |
|---|---|---|---|---|---|---|---|
| Be | 25.43 | 1.73(1.73[h]) | 2.47(2.47[h]) | 0.11 | 5.0 | 2 | 15.52 |
| Fe | 26.35(26.63[b]) | 1.91 | 2.16(2.20[h]) | -0.05 | 2.1 | 16 | 16.47 |
| Ni | 26.16(26.59[c]) | 1.91 | 2.34(2.35[h]) | 0.03 | 1.2 | 7 | 16.17 |
| Cr | 26.69(26.67[d]) | 1.97 | 1.91(2.06[d], 2.09[h]) | -0.17 | 3.8 | 30 | 17.02 |
| Co | 26.21 | 1.89 | 2.25(2.24[h]) | -0.01 | 0.5 | 11 | 16.28 |
| Cu | 26.58(26.34[e]) | 1.97 | 2.46(2.52[h]) | 0.09 | 3.6 | 0 | 16.33 |
| V | 26.90 | 2.01 | 2.09(1.97[h]) | -0.08 | 3.2 | 19 | 16.93 |
| Zn | 26.86(26.52[f]) | 2.03 | 2.56(2.64[h]) | 0.14 | 5.6 | 6 | 16.43 |
| Mo | 27.30(27.13[g]) | 2.08 | 2.11(2.14[h]) | -0.07 | 2.8 | 18 | 17.20 |
| Mn | 26.53 | 1.93 | 2.53(2.58[h]) | 0.13 | 5.5 | 5 | 16.24 |
| W | 27.21 | 2.06 | 2.19(2.21[h]) | -0.03 | 1.2 | 14 | 17.05 |
| Sn | 27.12 | 2.27 | 4.09(4.19[h]) | 0.91 | 31.4 | 93 | 15.14 |
| Ti | 26.66 | 1.96 | 2.81(2.87[h]) | 0.27 | 11.5 | 20 | 16.04 |
| Cd | 27.77 | 2.23 | 2.82 | 0.28 | 9.5 | 22 | 16.81 |
| Mg | 26.72 | 2.00 | 2.89(2.94[h]) | 0.31 | 12.4 | 25 | 16.00 |
| Sc | 27.04 | 2.07 | 3.08(3.15[h]) | 0.41 | 15.9 | 36 | 16.04 |
| Ca | 27.65 | 2.21 | 3.51(3.61[h]) | 0.63 | 22.7 | 61 | 16.03 |
| Sr | 28.15 | 2.39 | 3.80(3.90[h]) | 0.77 | 25.5 | 77 | 16.10 |
| Ba | 28.55 | 2.55 | 4.19 | 0.97 | 30.5 | 100 | 16.00 |

[a] distortion degree = $|d_M(M\text{-}4O) - d_{Cu}(M\text{-}4O)| / Max(d_M(M\text{-}4O) - d_{Cu}(M\text{-}4O))$; [b] Ref. [1]; [c] Ref. [2]; [d] Ref. [3]; [e] Ref. [4] ; [f] Ref. [5]; [g] Ref. [6], [h] Ref. [7]: VASP code, cutoff energy 500 eV, revPBE-vdW functional.

**3.1. NO molecular adsorption.**

When the Cu site is substituted by the alkaline earth metal (Be, Mg, Ca, Sr, and Ba), the NO adsorption via the t1 mode yields the adsorption energy $\Delta E_{ads}$ ranging between -16.0 and -28.7 kJ mol$^{-1}$. The adsorbed NO showed linear adsorption on M-HKUST-1 with the M-N-O angle of ~180 degree. Among them, Mg-HKUST-1 exhibits nearly the same ability with Cu-HKUST-1 with $\Delta E_{ads}$ of -28.7 kJ mol$^{-1}$, while the others show weaker chemisorptions compared with the Cu site. For the t2 mode configuration, Mg-HKUST-1 also yields the strongest adsorption with $E_{ads}$ of -16.7 kJ mol$^{-1}$ among all the alkaline earth metal substituted variants.

Of alternative twelve transition metals including Sc, Ti, V, Cr, Mn, Fe, Co, Ni, Zn, Mo, W, and Cd, there are six M-HKUST-1 (M = Ti, Mn, Sc, Ni, Co, and V) that have much larger adsorption energy in the absolute value than Cu-HKUST-1 for the t1 and t2 modes. Therefore, from the thermodynamic point of view, these alternative metal MOFs are expected to exhibit a better adsorption capacity than the original Cu-HKUST-1.

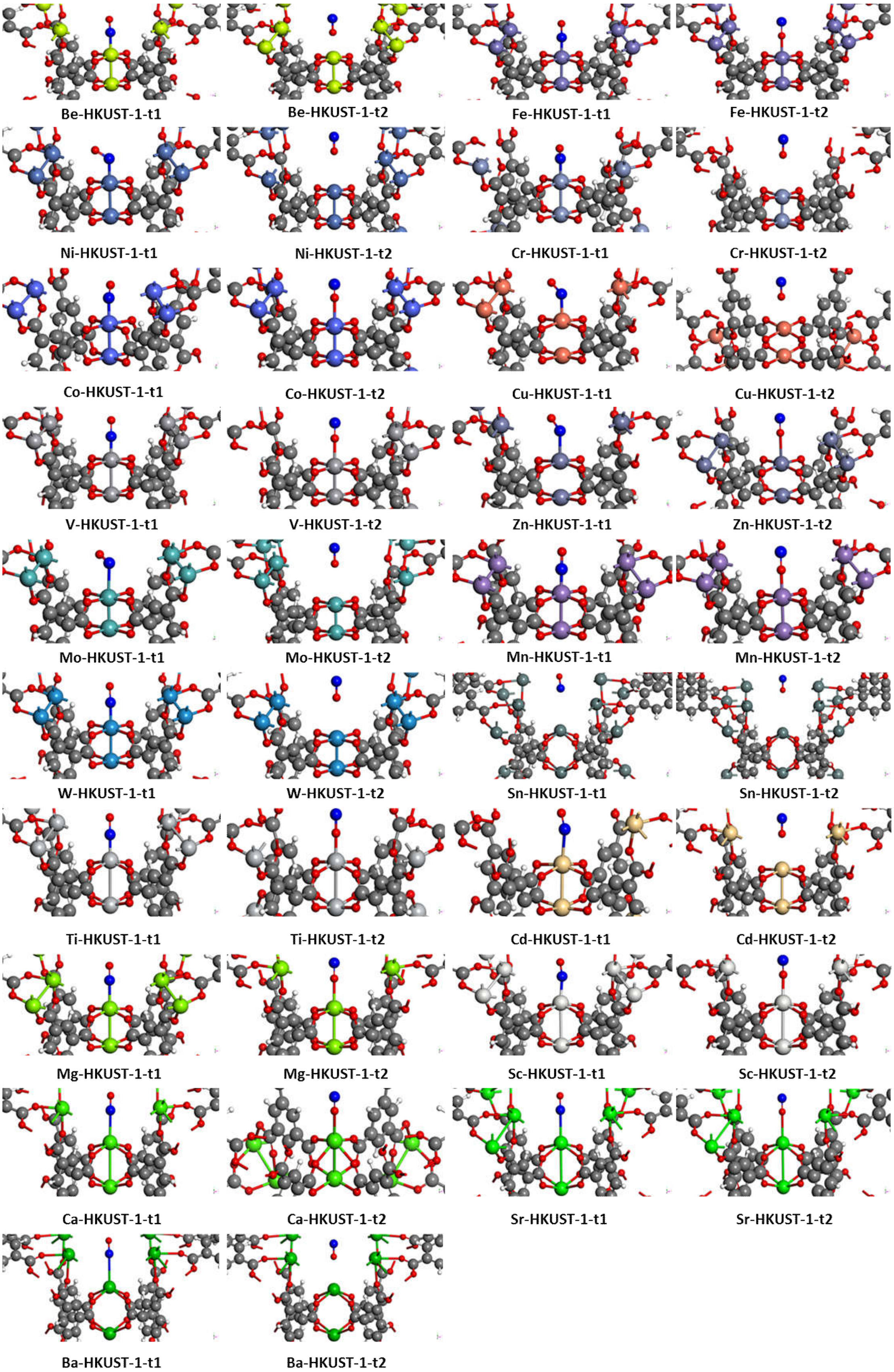


**Figure S2.** Adsorption configuration for NO molecular adsorption on M-HKUST-1. Atoms are shown in a sphere and stick form with white (H), red (O), gray (C), and blue (N) spheres. The largest spheres are metal atoms.

### 3.2. $NH_3$ molecular adsorption.

Of alternative twelve transition metals including Sc, Ti, V, Cr, Mn, Fe, Co, Ni, Zn, Mo, W, and Cd, there are three M-HKUST-1 variants (M = Co, Cd, Zn, and Ni ) yielding the lower adsorption energy (-95.2 to -128.7 kJ mol$^{-1)}$ than Cu-HKUST-1 (-79.8 kJ mol$^{-1}$) suggesting the better adsorption effect. V-HKUST-1 and Sc-HKUST-1 structures result in adsorption energies of -80.5 and -86.5 kJ mol$^{-1}$, which are close to Cu-HKUST-1. The other M-HKUST-1 materials exhibit the higher adsorption energy than Cu-HKUST-1.

Note that for $NH_3$ adsorption on M-HKUST-1(M = Mo, W, Sn, and Ba), in the initial structures, $NH_3$ bonds to the metal center via its N end with the d(M-N) bond distance of 1.85-2.20 Å like all the others, while after geometry optimization, it flipped over.

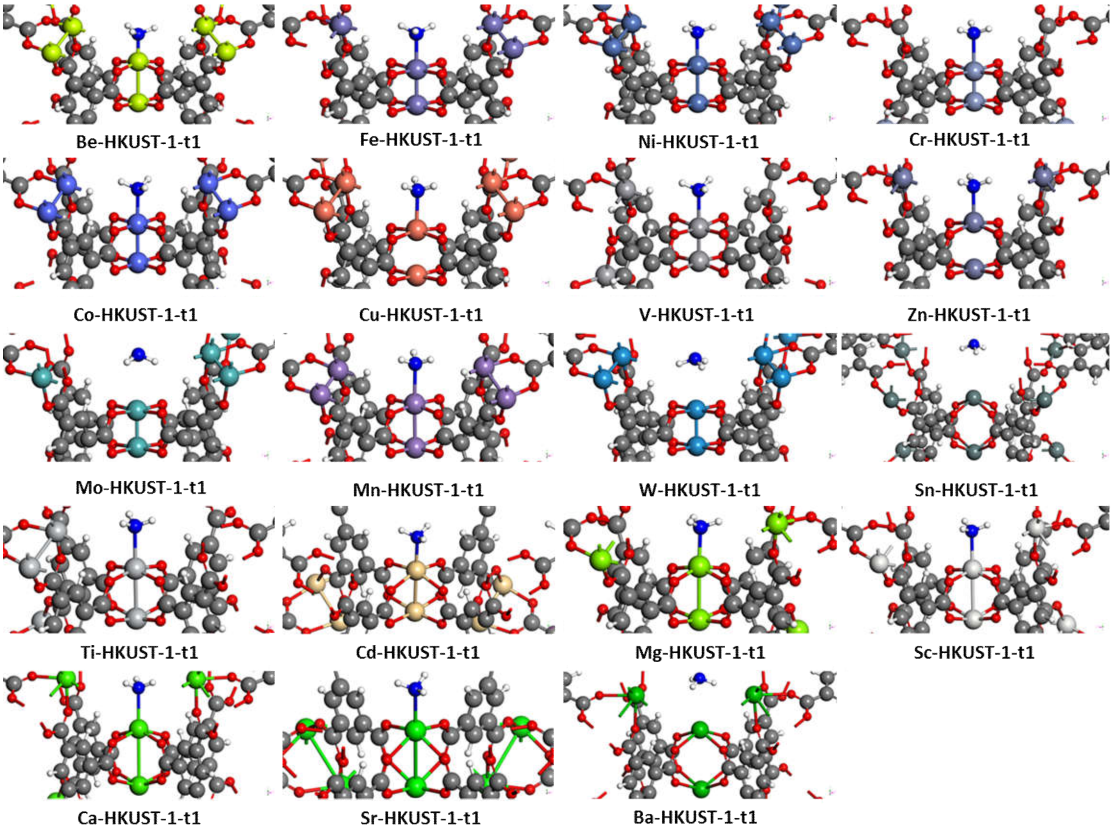


**Figure S3.** Adsorption configuration for $NH_3$ molecular adsorption on M-HKUST-1. Atoms are shown in a sphere and stick form with white (H), red (O), gray (C), and blue (N) spheres. The largest spheres are metal atoms.

**3.3. $NO_2$ molecular adsorption.**

The selected parameters for $NO_2$ molecular adsorption in M-HKUST-1 are summarized in Table S2. The most stable geometries on each metal center of M-HKUST-1 are provided in Figure S2.

When the Cu center of HKUST-1 is substituted by alkaline earth metal, similar to the case on the original Cu-HKUST-1, the most stable adsorption mode is still the t2 mode by forming one M-O bond. Among them, M-HKUST-1 (M = Ca, Sr, and Ba) exhibit the stronger $NO_2$ adsorption effect with the largest adsorption energy of ~ -44 kJ mol$^{-1}$, slightly larger than the value on Cu-HKUST-1 of -40.8 kJ mol$^{-1}$. Mg-HKUST-1 exhibits nearly the same $E_{ads}$ of -40.6 kJ mol$^{-1}$ with Cu-HKUST-1. Be-HKUST-1 yields a smaller $NO_2$ adsorption energy than Cu indicating a weaker $NO_2$ adsorption capacity than the original Cu-HKUST-1.

Of twelve transition-metal-substituted M-HKUST-1 (M = Sc, Ti, V, Cr, Mn, Fe, Co, Ni, Zn, Mo, W, and Cd), three M-HKUST-1 variants (M = Fe, Co, and Ni) yields the t1 mode by forming one M-N bond as the most stable adsorption configuration for $NO_2$ molecular adsorption. For M-HKUST-1 (M = Zn, Mo, Mn, W, and Cd), the most stable adsorption mode shifts to t2 by forming one M-O bond. For M-HKUST-1 (M = Cr, V, and Ti), the most stable adsorption mode is b2 by forming one M-N bond and one M-O bond. For Sc-HKUST-1 and Sn-HKUST-1, the most stable adsorption mode is b1 by forming two M-O bonds. However, the M-O bond distance on Sn-HKUST-1 is as large as ~2.91 Å yielding a higher adsorption energy of -14.5 kJ mol$^{-1}$, while the corresponding M-O bond distance on Sc-HKUST-1 is only 2.27 Å connecting with a much lower adsorption energy of -303.6 kJ mol$^{-1}$.

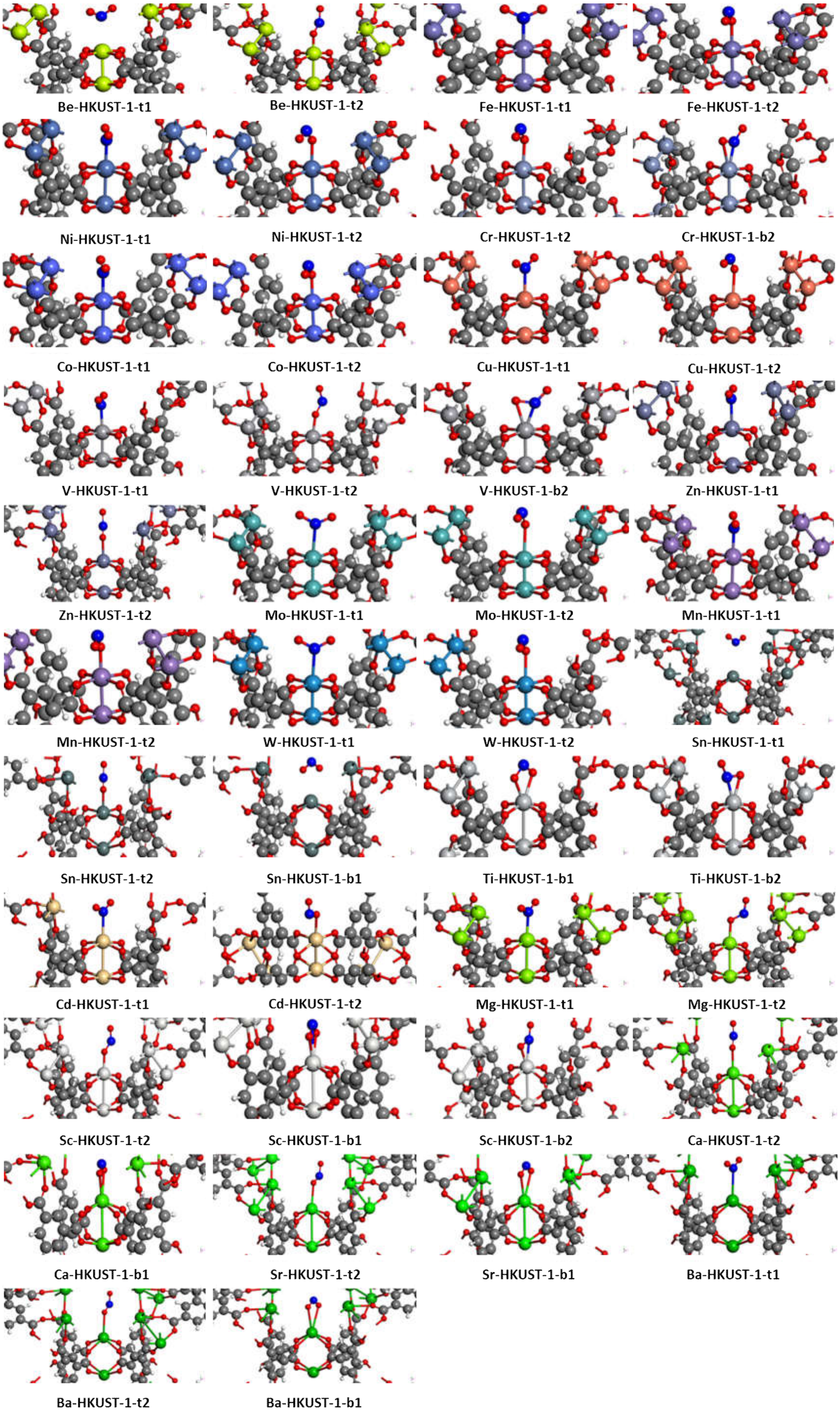
Be-HKUST-1-t1
Be-HKUST-1-t2
Fe-HKUST-1-t1
Fe-HKUST-1-t2
Ni-HKUST-1-t1
Ni-HKUST-1-t2
Cr-HKUST-1-t2
Cr-HKUST-1-b2
Co-HKUST-1-t1
Co-HKUST-1-t2
Cu-HKUST-1-t1
Cu-HKUST-1-t2
V-HKUST-1-t1
V-HKUST-1-t2
V-HKUST-1-b2
Zn-HKUST-1-t1
Zn-HKUST-1-t2
Mo-HKUST-1-t1
Mo-HKUST-1-t2
Mn-HKUST-1-t1
Mn-HKUST-1-t2
W-HKUST-1-t1
W-HKUST-1-t2
Sn-HKUST-1-t1
Sn-HKUST-1-t2
Sn-HKUST-1-b1
Ti-HKUST-1-b1
Ti-HKUST-1-b2
Cd-HKUST-1-t1
Cd-HKUST-1-t2
Mg-HKUST-1-t1
Mg-HKUST-1-t2
Sc-HKUST-1-t2
Sc-HKUST-1-b1
Sc-HKUST-1-b2
Ca-HKUST-1-t2
Ca-HKUST-1-b1
Sr-HKUST-1-t2
Sr-HKUST-1-b1
Ba-HKUST-1-t1
Ba-HKUST-1-t2
Ba-HKUST-1-b1

**Figure S4.** Adsorption configuration for $NO_2$ molecular adsorption on M-HKUST-1. Atoms are shown in a sphere and stick form with white (H), red (O), gray (C), and blue (N) spheres. The largest spheres are metal atoms.

**Table S2.** The bond distance and bond angle of $NO_2$ in the adsorption state. d1 and d2 (Å) are the N-O bond lengths in the $NO_2$ molecule and ang. (°) is the angle between the three atoms in the $NO_2$ molecule.

| | t1 | | | t2 | | | b1 | | | b2 | | |
|---|---|---|---|---|---|---|---|---|---|---|---|---|
| | d1 | d2 | ang. | d1 | d2 | ang. | d1 | d2 | ang. | d1 | d2 | ang. |
| Be | 1.21 | 1.21 | 134 | 1.25 | 1.21 | 125 | -- | -- | -- | -- | -- | -- |
| Fe | 1.24 | 1.24 | 125 | 1.30 | 1.23 | 121 | -- | -- | -- | -- | -- | -- |
| Ni | 1.23 | 1.23 | 128 | 1.27 | 1.22 | 125 | -- | -- | -- | -- | -- | -- |
| Cr | -- | -- | -- | 1.29 | 1.22 | 123 | -- | -- | -- | 1.26 | 1.22 | 127 |
| Co | 1.24 | 1.24 | 126 | 1.29 | 1.22 | 122 | -- | -- | -- | -- | -- | -- |
| Cu | 1.23 | 1.23 | 130 | 1.24 | 1.22 | 130 | -- | -- | -- | -- | -- | -- |
| V | 1.24 | 1.24 | 125 | 1.40 | 1.21 | 113 | -- | -- | -- | 1.29 | 1.22 | 125 |
| Zn | 1.23 | 1.23 | 128 | 1.26 | 1.21 | 124 | -- | -- | -- | -- | -- | -- |
| Mo | 1.24 | 1.24 | 126 | 1.27 | 1.23 | 123 | -- | -- | -- | -- | -- | -- |
| Mn | 1.24 | 1.24 | 125 | 1.35 | 1.22 | 118 | -- | -- | -- | -- | -- | -- |
| W | 1.25 | 1.24 | 124 | 1.30 | 1.23 | 119 | -- | -- | -- | -- | -- | -- |
| Sn | 1.22 | 1.22 | 132 | 1.42 | 1.20 | 111 | 1.24 | 1.23 | 125 | -- | -- | -- |
| Ti | -- | -- | -- | -- | -- | -- | 1.28 | 1.28 | 111 | 1.36 | 1.22 | 121 |
| Cd | 1.23 | 1.23 | 132 | 1.25 | 1.21 | 127 | -- | -- | -- | -- | -- | -- |
| Mg | 1.24 | 1.23 | 129 | 1.26 | 1.21 | 125 | -- | -- | -- | -- | -- | -- |
| Sc | -- | -- | -- | 1.37 | 1.22 | 113 | 1.28 | 1.28 | 112 | 1.32 | 1.22 | 119 |
| Ca | -- | -- | -- | 1.26 | 1.23 | 123 | 1.25 | 1.25 | 120 | -- | -- | -- |
| Sr | -- | -- | -- | 1.27 | 1.23 | 122 | 1.26 | 1.25 | 120 | -- | -- | -- |
| Ba | 1.25 | 1.25 | 124 | 1.26 | 1.24 | 122 | 1.26 | 1.25 | 120 | -- | -- | -- |

### 3.5. Charge Density Map

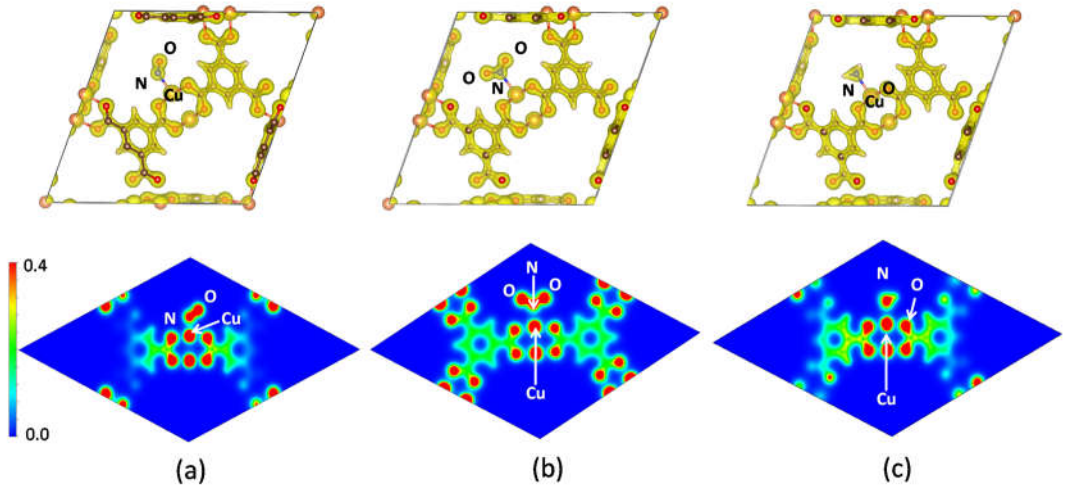


**Figure S5.** The 3D (top) and 2D (bottom) charge density map measured in e/Å3 for (a) NO, (b)$NO_2$, and (c) $NH_3$ adsorption on Cu-HKUST-1. The cutting plane for (a)-(c) is N-O-Cu where N-O are both from adsorbates, O-N-O, and N-Cu-O where O is from the MOFs and the bonded Cu, respectively.

**Reference**


1. Xie, L.; Liu, S.; Gao, C.; Cao, R.; Cao, J.; Sun, C.; Su, Z. Mixed-Valence Iron(II, III) Trimesates with Open Frameworks Modulated by Solvents. *Inorg Chem* **2007**, *46*, 7782–7788, doi:10.1021/IC062273M.
2. Maniam, P.; Stock, N. Investigation of porous ni-based metal-organic frameworks containing paddle-wheel type inorganic building units via high-throughput methods. *Inorg Chem* **2011**, *50*, 5085–5097, doi:10.1021/ic200381f.
3. Murray, L. J.; Dinca, M.; Yano, J.; Chavan, S.; Bordiga, S.; Brown, C. M.; Long, J. R. Highly-Selective and Reversible $O_2$ Binding in $Cr_3$(1,3,5-benzenetricarboxylate)$_2$. *J Am Chem Soc* **2010**, *132*, 7856–7857, doi:10.1021/ja1027925.
4. Chui, S. S.-Y.; Lo, S. M.-F.; Charmant, J. P. H.; Orpen, A. G.; Williams, I. D. A chemically functionalizable nanoporous material. *Science* **1999**, *283*, 1148–50, doi:10.1126/SCIENCE.283.5405.1148.
5. Feldblyum, J. I.; Liu, M.; Gidley, D. W.; Matzger, A. J. Reconciling the Discrepancies between Crystallographic Porosity and Guest Access As Exemplified by Zn-HKUST-1. *J Am Chem Soc* **2011**, *133*, 18257–18263, doi:10.1021/ja2055935.
6. Kramer, M.; Schwarz, U.; Kaskel, S. Synthesis and properties of the metal-organic framework $Mo_3$(BTC)$_2$. *J Mater Chem* **2006**, *16*, 2245–2248, doi:10.1039/b601811d.
7. Koh, H. S.; Rana, M. K.; Hwang, J.; Siegel, D. J. Thermodynamic screening of metal-substituted MOFs for carbon capture. *Phys Chem Chem Phys* **2013**, *15*, 4573–4581, doi:10.1039/c3cp50622c.
